# Assessment of nutritional status and associated factors among adolescent girls in Afar, Northeastern Ethiopia: a cross-sectional study

**DOI:** 10.1186/s41043-021-00227-0

**Published:** 2021-02-23

**Authors:** Gebrehiwot Hadush, Oumer Seid, Abel Gebre Wuneh

**Affiliations:** 1 Afar Region Health Bureau, Semera, Ethiopia; 2 School of Public Health, College of Health Sciences, Mekelle University, Mekelle, Ethiopia; 3 Department of Public Health, College of Medical and Health Sciences, Samara University, Samara, Ethiopia

**Keywords:** Adolescent girls, Prevalence, Thinness, Stunting, Northeast Ethiopia

## Abstract

**Background:**

A body of evidences showed that adolescent undernutrition is a serious public health problem in developing countries including Ethiopia. Adolescence period is the last chance for curbing the consequences of undernutrition and breaking the intergenerational cycle of malnutrition and poor health. Despite this fact, they have been considered as a low-risk group for poor health and nutrition problems than the young children or the very old. This study aimed to assess prevalence of nutritional status and associated factors among adolescent girls in Afar, Northeastern Ethiopia, 2017.

**Methods:**

A school-based cross-sectional study design was conducted among 736 adolescent girls from February15 to March 05, 2017 in Afar, Northeastern Ethiopia, 2017. Multi-stage sampling technique was used to select study participants. A pretested and structured interviewer-administered questionnaire and anthropometric measurements was used to collect the data. The collected data were entered in to Epi Data version 3.1 and exported to SPSS version 20.0 for further statistical analysis. Body Mass Index for age (thinness) and height for age (stunting) was used to assess undernutrition of adolescent girls by using the new 2007 WHO Growth Reference. Data were analyzed using bivariate and multivariable logistic regression. The degree of association between dependent and independent variables were assessed using odds ratio with 95% confidence interval, and variables with *p* value < 0.05 were considered significant.

**Results:**

The study revealed that the prevalence of thinness and stunting were 15.8% (95% CI 13.3–18.5%) and 26.6% (95% CI 23.5–29.9%), respectively. Being at an early adolescent age (AOR = 2.89, 95% CI 1.23–6.81) for thinness and being at an early adolescent age (AOR = 1.96, 95% CI 1.02–3.74), household food insecure (AOR = 2.88, 95% CI 1.15–7.21), menstruation status (AOR = 2.42, 95% CI 1.03–5.71), and availability of home latrine (AOR = 3.26, 95% CI 1.15–4.42) for stunting were the independent predictors among the adolescent girls.

**Conclusions:**

The prevalence of thinness and stunting is above the public health importance threshold level. Thus, Multi-sector-centered nutrition interventions to improve nutritional status of disadvantaged adolescent girls through providing comprehensive nutritional assessment and counseling services at community, school, and health facility levels, and creating household’s income-generating activities are recommended before they reach conception to break the intergenerational cycle effect of malnutrition.

## Background

World Health Organization (WHO) defined adolescence as a period of life ranging from 10 to 19 years old which is the transition from dependent childhood to independent adulthood [1]. Worldwide, there are about 1.2 billion adolescents, representing more than 18% of the global population. Nearly 90% of them live in developing countries, and approximately 600 million are female [2]. In Ethiopia, 20–26% of the population are adolescents [3].

Adolescence is a period of rapid growth and development by which up to 45% of skeletal growth takes place, and 15 to 25% of adult height is achieved [4]. Throughout this period, risk of nutrition inadequacies and other health issues are of great concern due to rapid growth in stature, muscle mass, and fat mass. As a result of these serious nutritional challenges, adolescents would be negatively affected by this rapid growth spurt as well as their health as adults [5].

Malnutrition in all its forms, particularly undernutrition including underweight for age, too short for age (stunted), too thin relative to height (wasted), and functionally deficient in vitamins and minerals, is a global issue, but in the developing countries, it is catastrophic [6, 7]. Adolescents are in a vulnerable group for malnutrition and its consequences, because it is a dynamic period of physical growth and mental development. Undernutrition starts before birth, goes into adolescence and adult life, and can span into generations and results in short stature, lean body mass, and is associated with deficiencies in muscular strength. In addition, it can reduce resistance to infection and other debilitating conditions that reduce productivity [8–10].

A body of evidence showed that globally, adolescent undernutrition is a serious public health problem in both developed and developing countries, but is disproportionally keeping sever in developing countries, especially in Asia (32–65%) and Africa (4–30%), making them more vulnerable to low productivity, poor health, and early deaths. In Sub-Saharan Africa, the prevalence of adolescent undernutrition is 15–58%, which is higher from other African countries [7, 11–13]. According to WHO, the recommended indicator to assess the nutrition status of adolescents are thinness (low body mass index for age) and stunting (low height for age), where the former is a result of mainly acute (short term), and the latter shows chronic (long term) nutritional deficiency [14–16].

Evidences showed that adolescent girls in many contexts are a marginalized and disempowered group and consequently face diminished opportunities and choices [17]. They are a nutritionally vulnerable group for their high requirements for growth, their eating patterns and lifestyles, and their risk-taking behaviors; their susceptibility to environmental influences and hard physical work, as commonly observed in low-income countries, may impose additional physiological stress and nutritional requirements in adolescence. In certain cultures, from infancy onwards including adolescents, girls are at particularly high risk because of gender discrimination [9, 18–20]. On top of this, adolescents have been considered a low-risk group for poor health and nutrition and often receive little attention. This results in lack of information regarding the nutritional status of adolescents especially from the developing world [20, 21].

There are few studies done in Ethiopia regarding the level of adolescent undernutrition in the country. The Ethiopian nutrition baseline report revealed that the prevalence of stunting and thinness in adolescent girls was 23 and 14%, respectively [22]. The other community-based studies done in different parts of Ethiopia such as Somali, Oromia, and Tigray indicated that the prevalence of both stunting and thinness were high in some rural parts of the country which were 22.9% stunted and 11.5% thin [23], 27.5% thin and 15.6% stunted [24], and 21.4% thin and 26.5% stunted [4], respectively.

Even though the few existing studies done on the nutritional status of adolescent girls in some parts Ethiopia and other developing world [10, 14, 19, 20] indicated that adolescent undernutrition is a major public health problem in developing countries including our country, data on adolescent girl’s nutritional status in pastoral societies are scarce. Despite the fact that having adequate evidences and information on nutritional status among adolescent girls do have a paramount step for intervention programs to break the intergenerational cycle of malnutrition, to the best of our knowledge, there was no previous study with this objective on adolescent girls in Afar regional state where pastoral communities live particularly in the study area. Therefore, this study aimed to assess the prevalence of nutritional status (thinness, stunting) and associated factors among adolescent girls in Megale district, Afar regional state, Northeastern Ethiopia.

## Methods

### Study design and setting

This study employed a school-based cross-sectional study design from February 15, to March 05, 2017 in selected schools found at Megale district, Afar National Regional State, North East Ethiopia. The district is located at a distance 325 km away to the west of the regional capital, Semera and 765 km northern east of the capital of Ethiopia, Addis Ababa. The district is typically rural and organized into 8 administrative kebeles (the smallest administrative units), and the community is characterized by pastoral livelihood.

According to the Megale district health office report, the total population of the district, in the year 2016, is estimated to be 34,692 (19,220 males and 15,473 females) and children aged 6 months to 59 years old are 3962. Currently, the district has 21 governmental primary schools in the academic year of 2016/2017. The district has 3 health centers, 7 health posts, one private drug store, and one pharmacy. The topography is 60% mountainous, 20% flat, and 20% inclined. The annual rainfall is 500–600 ml, and the temperature is 35–40 °C [25].

### Study populations

All adolescent girls (10–19 years) found in the governmental primary school of Megale district were the targets for the study, where the study population consisted of a sample of all regular adolescent girls found residing in the randomly selected governmental primary schools during the study period. Those adolescent girls who had physical deformity that hinder height measurements, self-reported pregnancy status, and residents of the study area for less than 6 months in the family at the time of interview were excluded from the study.

### Sample size and sampling procedure

The required sample size for the first objective of this study (to determine the prevalence of thinness and stunting) was determined using a single population proportion with the following assumptions: The level of confidence (*α*) 95% (*Z*1-α /2 = 1.96), margin of error (*d*) 5%, design effect of 2 and the proportions (*p*) of adolescents’ girls who had thinness and stunting were 22.9 and 11.5% respectively taken from previous study done in Somalia region, Ethiopia [23], and the higher prevalence (22.9%) was taken and calculated using *z*2 × *p* × *q*/*d*^2^. Therefore, the final sample size by considering the non-response rate of 10% was 298.

The required sample size for the second objective of this study (for the factors associated with thinness and stunting) was determined using Open Epi menu online software program with the following assumptions: The level of confidence (α) is taken to be 95%, power 80; and ratio (unexposed: exposed) was taken only once (Table 1).

**Table 1:** Sample size determination using Open Epi menu online software program for each factor significantly associated with the outcome variables found from different literatures, 2017

Factors considered	Percent of exposed and non-exposed with outcome variable	Final sample size (with10% non-response rate)	References taken
Income status	Thinness among adolescent girls with higher family income (15.6%) and lower family income (28.8%)	334 + 334 × 10% = 369	[23]
Dietary diversity	Stunting among adolescent girls with adequate dietary diversity score (54.1%) and inadequate dietary diversity score (71.2%)	272 + 272 × 10% = 299	[24]
Income status	Stunting among adolescent girls with higher family income (6.4%) and lower family income (16.6%)	344 + 344 × 10% = 379	[23]
Diarrhea illness	Thinness among adolescent girls with diarrhea illness in the last 2 weeks (44.6%) and with no diarrhea illness (20.1%)	128 + 128 × 10% = 141	[24]
Menarche started	Stunting among adolescent girls who started menarche (< 14 years) (21.9%) and did not start menarche (> 14 years) (37.3%)	300 + 300 × 10% = 330	[8]

At the end, out of two objectives, the prevalence (290) and associated factors (379), the largest sample 379 was used for this study. Considering a design effect of 2 (379 × 2 = 758), the final sample size calculated was 758.

Study participants were selected by multistage random sampling method. First, out of the twenty one governmental primary schools (grade 4–grade 8), eight schools were selected randomly. Second, the total sample size was allocated in to each randomly selected school using proportion to population size (PPS). Finally, after taking a list of an identification number for each adolescent girl student in the randomly selected schools from each school’s administrators (from their roster) as a sampling frame (list of students between 10 and 19 years), study participants were selected using simple random sampling technique randomly by computer-generated random numbers.

### Data collection tools and process

A structured questionnaire was developed from the Ethiopian national nutrition survey report for the national nutrition program of Ethiopia [22] and other relevant literatures and contextualized to the local situation. The questionnaire was composed of sociodemographic and economic factors, health- and environment-related factors, dietary habits, and anthropometrics. Concerning the dietary diversity, individuals were asked about their past 24-h dietary recall method (from sunrise to sunrise), while for the dietary food frequency, individuals were asked about their past 7 days of food frequency practice using the WHO nine food groups. The minimum dietary diversity score of four or more out of the nine groups of foods was considered as adequate [26].

Anthropometric measurements such as body weight and height were measured, the former by using a weighing scale in light clothing with no jackets or coats, shoes, and additional clothing to the nearest 0.1 kg on a new calibrated portable scale and the latter by using a portable stadiometer with no shoes; shoulders, buttocks, and heels touching the vertical stand; and the head in Frankfurt position to the nearest 0.1 cm, respectively. Mid upper arm circumference (MUAC) was measured by marking midway between shoulder tip and the elbow tip on the vertical axis of the upper arm with the arm bent at right angle and between the lateral and medial surface of the left arm. Four diploma female nurses as data collectors and two BSc nursing professionals as supervisors were recruited. For each participant from the eight primary schools, direct face-to-face interviews were conducted during their break time before noon.

### Data quality control

English version questionnaire was translated into the local language, “Afaraff”, and then back to English to maintain its consistency. Pretest was conducted among 37 students (5% of the sample) in a non-selected school in the district for necessary modification. A two-day training was given to the data collectors and supervisors before the actual data collection. Continuous supervision was done by the supervisors and the principal investigator on a daily basis.

### Statistical analysis

All raw data with the exception of anthropometric data were entered and cleaned in EPI data software version 3.1 and then exported to SPSS for analysis; whereas the anthropometric data were entered and converted to height-for-age and BMI-for-age *Z* scores by using the Antro Plus software. Adolescent girls with BMI-for-age below −2*Z* scores and height-for-age below −2*Z* scores of the 2007 WHO reference population were classified as thin and stunted, respectively [27]. Descriptive statistical measures such as percentage, mean, and standard deviation of variables were computed to summarize the data.

Binary logistic regression model was used to assess the association between the two dependent and independent variables using odds ratio with 95% confidence interval. To identify independent variables which have statistically significant association with the outcome variable (thinness and stunting), first, bivariate analysis was computed for each independent variable, and the outcome variables and crude odds ratio (COR) and 95% confidence interval (CI) were obtained.

Then, all variables observed to be significant in the bivariate logistic analysis (at *p* value < 0.25) were subsequently included in the multivariable logistic regression model to identify the independent predictor variable after controlling the effects of confounders and adjusted odds ratio (AOR) with 95% CI was calculated. Multicollinearity between the independent variables was checked using standard error and excluded the variables that had standard error of > 2, and Goodness of fit was checked by the Hosmer & Lemeshow test with *p* value > 0.05. All tests were two-sided, and *p* values of less than 0.05 were considered to be predictive for each outcome variable. Results were described and presented using narrative text, graphs, and tables.

### Operational definitions

Adolescents are individuals in the age group of 10–19 years of age. It is categorized as early (adolescents in the age group of 10–13 years of age), middle (adolescents in the age group of 14–16 years of age), and late adolescents (adolescents in the age group of 17–19 years of age) [28].

Stunting is if the height-for-age *Z* score is found to be below −2 SD of the 2007 WHO growth reference. Severe stunting is diagnosed if it is below −3 SD [27, 29].

Thinness is if the BMI-for-age *Z* score < −2 SD of the WHO growth reference 2007. Severe thinness is diagnosed if it was below −3 SD [27, 29].

Body mass index (BMI) is defined as weight in kilograms divided by height in meters squared = Weight (kg)/Height (m^2^)—normal weight if 18.5 kg/m^2^ < BMI < 25 kg/m^2^, underweight if BMI < 18.5 kg/m^2^, and overweight if BMI > 25 kg/m^2^ [27, 29].

Mid upper arm circumference (MUAC) < 18 cm is classified as severe acute malnutrition, MUAC of 18–21 as moderate acute malnutrition, and MUAC > 21 is classified as normal [27, 29].

Household food security was assessed using the four-item module, and the sum of affirmative responses to the six questions in the module was taken. The food security status of households with raw score 0–1 was described as food secure and food insecure [23].

Adequate dietary diversity score is defined as adolescent girls with dietary diversity score of the median and above the median values (> 4 food groups), whereas inadequate dietary diversity score is when adolescent girls with dietary diversity score is below the median value (< 4 food groups) [26].

### Ethical considerations

Ethical clearance was obtained from Mekelle University, College of Health Sciences, Research and Community Service Unit Ethical Review Committee. A support letter was also obtained from Afar regional education Bureau, Megale district health and education offices and kebele administrations. Again, informed consent was obtained from the commandant of the schools, participant, participant’s parent/ guardian before being enrolled, and they were assured about the confidentiality of the information. The aims of study and any possible risk of the study were explained to study participants using their own local language.

## Results

### Demographic and socioeconomic characteristics

A total of 736 adolescent girls participated in this study with a response rate of 97.4%. The mean ± SD age of study participants were 14.28 ± 2.79 years where around two fifth, 286 (38.9%), of them were in the early adolescence period, while 178 (24.2%) were in the late adolescence period. Slightly below three fourths, 270(73.4%), of participants were rural residents. Majority, 714 (97.0%) and 712 (96.7%), of the participants were Muslims in religion and Afar in ethnicity, respectively. Moreover, 628(85.3%) of them were single, while the remaining 108 (14.7%) were currently married (Table 2).

**Table 2:** Demographic and socioeconomic-related characteristics of school’s adolescents girls in Megale district, Afar Regional state, Northeastern Ethiopia, April, 2017 (*n* = 736)

Variables	Categories	*N*	%
Age of adolescent (in years)	Early adolescent (10–13)	286	38.8
	Middle adolescent (14–16)	272	37.0
	Late adolescent (17–19)	178	24.2
Adolescent residence	Rural	540	73.4
	Urban	196	26.6
Adolescent religion	Muslim	714	97.0
	Orthodox	22	3.0
Adolescent ethnicity	Afar	712	96.7
	Tigray	24	3.3
Adolescent marital status	Unmarried	628	85.3
	Married	108	14.7
Grade level of respondents	4	266	36.1
	5	168	22.8
	6	102	13.9
	7	64	8.7
	8	136	18.5
Head of house hold	Male	702	95.4
	Female	34	4.6
Family size	< 5	96	13.0
	5–10	582	79.1
	> 10	58	7.9
Occupation status of father	Farmer	54	7.4
	Pastoralist/herding livestock	514	69.8
	Government employee	168	22.8
Occupation status of mother	Housewife	672	91.3
	Pastoralist/herding livestock	6	0.8
	Government employee	58	7.9
Education status of father	No formal education	674	91.6
	Primary school (1–8)	16	2.2
	Secondary & preparatory school	34	4.6
	College and above	12	1.6
Education status of mother	No formal education	704	95.7
	Primary School (1–8)	18	2.4
	Secondary & preparatory school	14	1.9
Family monthly income (ETB)	< 500	474	64.4
	500–1000	156	21.2
	> 1000	106	14.4
Source of food	Own production	34	4.6
	Purchased	558	75.8
	Food aid	144	19.6
Farming land ownership	Yes	54	7.3
	No	682	92.7
Availability of garden near home	Yes	46	6.2
	No	690	93.8
Availability of livestock	Yes	714	97.0
	No	22	3.0
Household ownership of assets^a^	Camels	516	72.5
	Oxen	44	6.2
	Cows	58	7.9
	Goats	684	95.8
	Sheep	528	73.9
	Donkeys	480	67.4

^a^ Multiple responses in %, *ETB* Ethiopian Birr

The educational distribution of the students’ parents showed that 704 (95.7%) and 674 (91.6%) of their mothers and fathers did not attend formal education, whereas the least percent have joined college or university, 14(1.9%) for mothers and 12 (1.6%) for fathers. Regarding the occupation of parents, majority of the fathers’ occupation were pastoral/herding livestock, 514 (69.8%) and followed by government employee, 168 (22.8%). Majority of mothers’ occupation were housewife, 672 (91.3%) and followed by government employee, 58 (7.9%). Around 702 (95.4%) of households were headed by males and 34 (4.6%) were by females (Table 2).

### Health and household environment-related characteristics

Slightly below one third, 168 (22.8%) of the participants reported that they have a home latrine, and 162 (96.4%) of them were using a latrine. Concerning school latrine utilization, 154 (20.9%) of the participants do not use the school latrine. Regarding the source of drinking water, 276 (37.5%) of them obtained from a protected or safe water source. Again in terms of waste disposal method, 702 (95.4%) of the participants use the open-field waste disposal method (Table 3). About 206 (28.0%) of adolescent girls started menstruation, and the mean ± SD age of menarche was 13.86 + 1.84 years. Moreover, 112 (15.2%) had history of illness in the past 2 weeks prior to the data collection.

**Table 3:** Health and household environment-related characteristics of school adolescents girls in Megale district, Afar Regional state, Northeastern Ethiopia, April, 2017 (*n* = 736)

Variables	Categories	*n*	%
Availability of home latrine	Yes	168	22.8
	No	568	77.2
Type of home latrine	Pit latrine	168	100
Home latrine utilization	Yes	162	96.4
	No	6	3.6
Availability of school latrine	Yes	736	100
School latrine utilization	Yes	582	79.1
	No	154	20.9
Availability of water & soap/ ash near home latrine for hand washing	Yes	16	2.2
	No	720	97.8
Hand washing with soap/ ash after latrine utilization	Yes	178	24.2
	No	558	75.8
Source of drinking water	Protected	276	37.5
	Unprotected	460	62.5
Waste disposal method	Open field	702	95.4
	Pit	34	4.6
Menstruation started	Yes	206	28.0
	No	530	72.0
History of illness in the last 2 weeks	Yes	112	15.2
	No	624	84.8

### Dietary intake-related characteristics

#### Eating behavior and dietary diversity score of adolescent girls

Based on the 24-h dietary recalls, the overall proportion of adolescent girls with minimum dietary diversity score (at least consumed four food groups out of nine food groups) was 98 (13.3%). The dietary diversities consumed out of nine food groups were 640 (87.0%), 80 (10.9%), and 16 (2.1%), for low, medium, and high scores, respectively (Table 4).

**Table 4:** A 24-h recall dietary diversity practice of school adolescent girls in Megale district, Afar Regional state, Northeastern Ethiopia, April, 2017 (*N* = 736)

Food type or group	Categories	*N*	%
Do you skip any regular meal in the past two weeks?	Yes	134	18.2
	No	602	81.8
Types of meal skipped (*n* = 134)	Breakfast	108	80.6
	Lunch	8	6.0
	Dinner	18	13.4
Do you eat snacks between meals?	Yes	464	63.0
	No	272	37.0
Did you eat breakfast today (during the day of interviewing)?	Yes	698	94.8
	No	38	5.2
Did you get nutritional education in the past two weeks?	Yes	58	7.9
	No	680	92.1
Source of information	School	26	44.8
	Television	8	13.8
	Radio	12	20.7
	Textbook	4	6.9
	Nurse	8	13.8
The dietary diversities score (DDS) consumed out of nine food groups	Low (< 4)	638	86.7
	Medium (4–5)	82	11.2
	High (6–9)	16	2.2
Dietary diversities score (DDS)	DDS < 4	638	86.7
	DDS ≥4	98	13.3

**Fig. 1:**
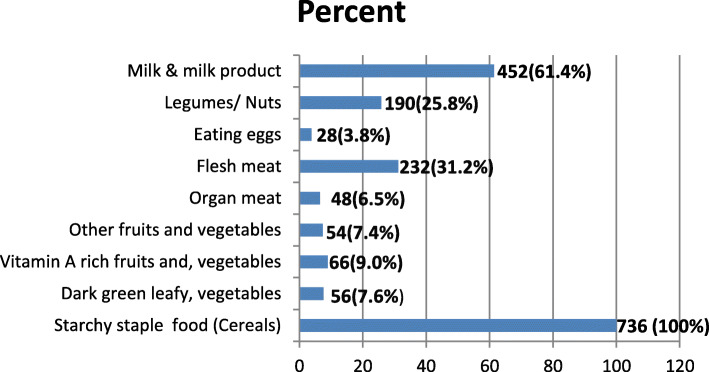
Types of food groups consumed over a 24-h period by school adolescents girls in Megale district, Afar Regional state, Northeastern Ethiopia, April, 2017 (*n* = 736)

Among the participants, 736 (100 %) consumed starchy staple food (cereals) followed by milk & milk products 452 (61.4%), flesh meat 232 (31.5%), and legumes/nuts 190 (25.8%). Consumption of dark green leafy vegetables, vitamin a-rich fruits and vegetables, and animal source foods (like organ meat, others fruits and vegetables, and eggs) were relatively low (Fig. 1).

#### Past 7-day food frequency of adolescent girls

Based on the 7-day food frequency report, 736 (100%) of them consumed starchy staple food (cereals), three or more times per week, followed by milk & milk products 604 (82.1%) and legumes/nuts 330 (44.8%), whereas dark-green leafy vegetables, vitamin-A rich fruits and vegetables, and animal source foods (like organ, flesh meat, & eggs) were relatively least consumed (Table 5).

**Table 5:** A 7-day food frequency of school adolescents girls in Megale district, Afar Regional state, Northeastern Ethiopia, April, 2017 (*n* = 736)

Variables	Categories	*N*	%
Starchy staple food (cereals)	≥ 3times per week	736	100.0
Vitamin A-rich fruits and vegetables	Never ate	293	79.6
	1–2 times per week	70	19.0
	≥ 3 times per week	5	1.4
Other fruits and vegetables	Never	257	69.8
	1–2 times per week	94	25.5
	≥ 3 times per week	17	4.6
Dark-green leafy vegetables	Never	256	69.6
	1–2 times per week	102	27.7
	≥ 3 times per week	10	2.7
Legumes/nuts	Never	137	37.2
	1–2 times per week	66	17.9
	≥ 3 times per week	165	44.8
Eggs	Never	308	83.7
	1–2 times per week	32	8.7
	≥3 times per week	28	7.6
Flesh meat	Never	143	38.9
	1–2 times per week	148	40.2
	≥ 3 times per week	77	20.9
Organ meat	Never	186	50.5
	1–2 times per week	116	31.5
	≥ 3 times per week	66	17.9
Milk & milk products (whole milk, cheese, yogurt)	Never	46	12.5
	1–2 times per week	20	5.4
	≥ 3 times per week	302	82.1

#### Prevalence of thinness and stunting of adolescent girls

**Fig. 2:**
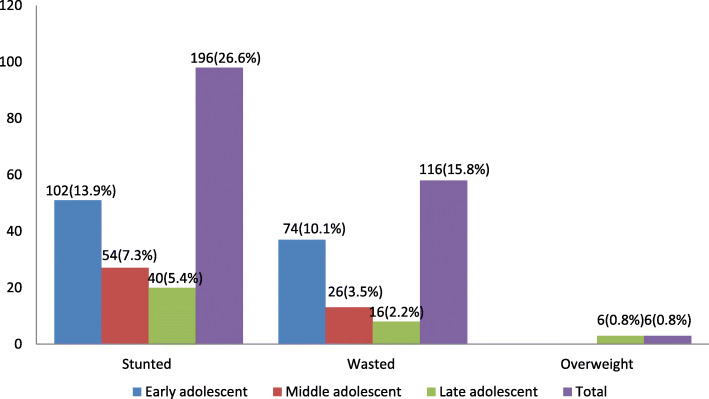
Overall anthropometric status of school adolescent girls in Megale district, Afar Regional state, Northeastern Ethiopia, April, 2017 (*N* = 736)

The mean ± SD overall height and weight of the participants was 145.8 ± 10.3 cm and 39.1 ± 9.3 kg, respectively. In this study, the overall prevalence of thinness (BAZ < − 2 SD) was 116 (15.8%) (95% CI 13.3–18.5%), the overall prevalence of stunting (HAZ < − 2 SD) was 196 (26.6%) (95% CI 23.5–29.9%), while the prevalence of overweight was 6 (0.8%). The prevalence of severe thinness (BAZ < − 3 SD) and stunting (HAZ < − 3 SD) were 3.8 and 7.6%, respectively. The nutritional status of the adolescent girls according to the body mass index (BMI) showed that 198 (26.9%) of them were underweight. Moreover, according to their mid upper arm circumference (MUAC), 336 (45.7%) of the adolescent girls were found to have moderate acute malnutrition (MUAC 18–21 cm) (Table 6).The anthropometric measurements indicated that early age of adolescent girls were more stunted 102 (13.9%) and thin 74 (10.1%) than late adolescents 40 (5.4%) and 16 (2.2%), respectively (Fig.2).

**Table 6:** Anthropometric status of study participants of school adolescent girls in Megale district, Afar Regional state, Northeastern Ethiopia, April, 2017 (*N* = 736)

Variables	Categories	***n***	%
Height for age (stunting)	Severe stunting	56	7.6
	Moderate stunting	140	19.0
	Not stunted	540	73.4
BMI (cm)	Underweight (< 18.5)	198	26.9
	Not underweight ( ≥ 18.5)	538	73.1
BMI for age (thinness)	Severely thin	28	3.8
	Moderately thin	88	12.0
	Normal	614	83.4
	Overweight	6	0.8
MUAC (cm)	< 18	128	17.4
	18–21	336	45.7
	> 21	270	36.7

### Factors associated with thinness and stunting of adolescent girls

#### Factors associated with thinness

In the first logistic regression model, the variables significantly associated with adolescent girls’ thinness were being early adolescent age, eating snacks, grade level, marital status, menarche, and dietary diversity have association at *p* value < 0.25. In the final multivariable analysis after examining the effect of confounders, the independent predicators for thinness were being early adolescent age (AOR = 2.89, 95% CI 1.23–6.81). The odds of thinness were around 2.89 times higher among adolescent girls who were early adolescent girls than those who were late adolescents. However, the other determinant factors did not show an association with thinness in multivariable analysis (Table 7).

**Table 7:** Bivariable and multivariable logistic regression predictors of thinness among school adolescent girls in Megale district, Afar Regional state, Northeastern Ethiopia, April, 2017 (*N* = 736)

		Thinness			
Variables	Categories	Yes (%)	No (%)	COR (95% CI)	AOR (95% CI)
Age of adolescents	Early (10–13)	74 (25.9)	212 (74.1)	3.53 (1.56–8.00)	2.89 (1.23–6.81)*
	Middle (14–16)	26 (9.6)	246 (90.4)	1.07 (0.43–2.69)	0.99 (0.39–2.57)
	Late (17–19)	16 (9)	162 (59.6)	1	1
Eating snacks	No	46 (16.9)	226 (83.1)	1.15 (0.65–2.04)	0.93 (0.49–1.67)
	Yes	70 (15.1)	394 (84.9)	1	1
Grade level	4th	50 (18.8)	216 (81.2)	2.92 (1.06–8.00)	2.27 (0.71–7.28)
	5th	38 (22.6)	130 (77.4)	3.68 (1.29–10.47)	2.25 (0.68–7.49)
	6th	12 (11.8)	90 (88.2)	1.68 (0.48–5.85)	0.99 (0.25–3.99)
	7th	6 (9.4)	58 (90.6)	1.30 (0.29–5.83)	1.52 (0.30–7.59)
	8th	10 (7.4)	126 (92.64)	1	1
Marital status	Unmarried	108 (17.2)	520 (82.8)	2.59 (0.90–7.49)	1.05 (0.28–3.92)
	Married	8 (7.4)	100 (92.6)	1	1
Menarche	No	100 (19.0)	430 (81.0)	2.76 (1.26–6.05)	0.53 (0.18–1.53)
	Yes	16 (7.8)	190 (92.2)	1	1
Dietary diversity score	DDS < 4	108 (16.3)	530 (83.7)	2.29 (0.79–6.64)	0.44 (0.14–1.38)
	DDS ≥ 4	8 (12.2)	90 (87.8)	1	1

*p value < 0.05, *DDS* dietary diversity score, *OR* odds ratio, *AOR* adjusted odds ratio, *CI* confidence interval

#### Factors associated with stunting

In the first logistic regression model, the variables significantly associated with adolescent girls’ stunting were being early adolescent age, menarche, availability of home latrine, household food insecurity, grade level, family monthly income level, dietary diversity, source of water, and eating snack. Finally, those variables were taken to the final multivariable logistic regression to identify the variables significantly associated with stunting after controlling the effect of confounders. Hence, in the multivariable logistic regression analysis models, being early adolescent age (AOR = 1.96, 95% CI 1.02–3.74), household food insecure (AOR = 2.88, 95% CI 1.15–7.21), menstruation status (AOR = 2.42, 95% CI 1.03–5.71), and availability of home latrine (AOR = 3.26, 95% CI 1.15–4.42) were the independent predictors for stunting.

The odds of stunting were around 1.96 times higher among adolescent girls who were early adolescent girls than those who were of late adolescent age. Those adolescent girls whose households were food insecure were around 2.88 times more likely to get stunted as compared with those whose households were food secure, and those who had not had home latrine were 3.26 times more likely to get stunted as compared with those who had home latrine. Adolescent girls who did not start menstruation were 2.42 times more likely to be stunted as compared with adolescent girls who started menstruation (Table 8).

**Table 8:** Bivariable and multivariable logistic regression predictors of stunting among school adolescent girls in Megale district, Afar Regional state, Northeastern Ethiopia, April, 2017 (*N* = 736)

		Stunting			
Variables	Categories	Yes (%)	No (%)	COR ( 95% CI)	AOR (95% CI)
Age in year	Early(10–13)	102 (35.7)	184 (64.3)	1.91 (1.05–3.49)	1.96 (1.02–3.74)*
	Middle(14–16)	54 (20)	218 (80)	0.86 (0.45–1.64)	0.75 (0.38–1.49)
	Late(17–19)	40 (22.5)	138 (77.5)	1	1
Menarche started	No	162 (30.1)	368 (69.4)	2.23 (1.24–3.99)	2.42 (1.03–5.71)*
	Yes	34 (16.5)	172 (83.5)	1	1
Availability of home latrine	No	164 (28.9)	404 (71.1)	1.73 (0.95–3.15)	3.26 (1.15–4.42) *
	Yes	32 (19)	136 (81)	1	1
Household food insecure	Yes	22 (47.8)	24 (52.2)	2.72 (1.16–6.38)	2.88 (1.15–7.21)*
	No	174 (25.2)	516 (74.8)	1	1
Grade level of female students	4th	64 (24.1)	202 (75.9)	1.22 (.060–2.49)	0.51 (0.22–1.20)
	5th	68 (40.5)	100 (59.5)	2.62 (1.26–5.45)	1.18 (0.49–2.80)
	6th	18 (17.6)	84 (82.4)	0.827 (0.33–2.09)	0.37 (0.13–1.07)
	7th	18 (28.1)	46 (71.9)	1.51 (0.57–3.98)	1.23 (0.42–3.60)
	8th	28 (20.6)	108 (79.4)	1	1
Source of water	Unprotected	132 (28.7)	328 (71.3)	1.33 (0.82–2.17)	0.38 (0.18–3.83)
	Protected	64 (23.2)	212 (76.8)	1	1
Eating snacks	No	88 (32.4)	184 (67.6)	1.58 (0.98– 2.53)	1.21 (0.71–2.07)
	Yes	108 (23.3)	356 (76.7)	1	1
Family monthly income level (ETB)	< 500	136 (28.7)	338 (71.3)	0.930 (0.49–1.78)	0.63 (0.29–1.39)
	500–1000	28 (17.9)	128 (82.1)	0.51 (0.22–1.15)	0.43 (0.17–1.05)
	> 1000	32 (30.2)	74 (69.8)	1	1
Dietary diversity score	DDS < 4	172 (27.0)	466 (73.0)	1.38 (0.99–2.51)	0.34 (0.15–3.22)
	DDS ≥ 4	24 (24.5)	74 (75.5)	1	1

*p value < 0.05, *ETB* Ethiopian Birr, *DDS* dietary diversity score, *OR* odds ratio, *AOR* adjusted odds ratio, *CI* confidence interval

## Discussions

Adolescents have specific health and development needs, and many of them face challenges that hinder their well-being especially on adolescent girls such as adverse reproductive outcomes, pregnancy outcomes, and birth weight [26, 30]. Despite this fact, many studies in Ethiopia are still carried out focusing on the vulnerable groups like infant, pregnant and lactating women, and limited on adolescent girls. Hence, this study aimed to assess prevalence of nutritional status and associated factors among adolescent girls in primary schools of Megale district, Afar region, North East Ethiopia.

This study revealed that the overall prevalence of thinness among the adolescent girls was 15.8% (95% CI 13.3–18.5%), and this finding is almost similar using the same cutoff point with study done in Asembo and Mumias, Kenya (15.6%) [31], Kavre District, Nepal (14.94%) [32], Burkina Faso (13.7%) [33], and west Bengal (16%) [34]. It is consistent with the prevalence reported in Addis Ababa (13%) and Mekele (14%) [26, 35] but lower than the study done in Adwa town (21.4%) [4], Ambo (27.5%) [36], and Eastern Tigray, Ethiopia (33.7%) [8]. Again, it is much lower when we compared with the study done in Kolar District, Garhwal, India, rural community of Tigray, Ethiopia, and Northern Nigeria where 54.8, 43.47, 58.3, and 58.7% of the adolescent girls were thin [30, 37–39], respectively, but higher than study conducted in Tamale Metropolis, Ghana (10%) [40].

Other studies conducted in Addis Ababa city, Ethiopia (6.2%) [41] and Tunisia (1.3%) [42] have been reported much lower prevalence than the current study. These findings indicated that thinness is a major public health problem in majority of Ethiopian and other communities. The possible explanation for this difference could be due to difference in the study group and urban–rural difference between the study subjects and settings. Unlike this study, some studies done in Tunisia considered adolescents the middle and late stages which are less likely to be thin because of less possibility of height growth than early adolescents. The other possible variation could also be due to socioeconomic and cultural difference in dietary habit and care practices of study populations.

The overall prevalence of stunting in this study was also found to be 26.6% (95% CI 23.5–29.9%). This finding was consistent with other studies done in the rural community of Tigray, Ethiopia which reported that prevalence of stunting were 26.5% [39]. It is also consistent with study done in Nepal (21.08%) [32] and Seychelles (23%) [43]. Other previous studies in adolescent Ethiopians girls also reported that much lower levels of stunting. These include studies in Somali, Ethiopia (11.5%) [23], Adama zone (15.6%) [24], and Adwa Ethiopia (12.1%) [4].

Nonetheless, in northern Ethiopia, the prevalence of childhood chronic malnutrition is very high which may have an impact on the level of adolescent stunting [44]. A number of studies in other African countries including Burkina Faso (8.8%) [33] and Kenya (12.1%) [31] have been reported a lower prevalence of stunting. However, a high prevalence of stunting in adolescent girls has been reported in Bangladesh (32%) [45] and Garhwali, India (30.43%) [30].The variation could be due to socioeconomic and cultural difference in food access, nutrition information, dietary habit, and care practices of the communities.

In this study among the variables moved to the final multivariable logistic regression analysis model, being of early adolescent age was found to be the independent predictor for thinness. Hence, the odds of thinness were around 2.89 times higher among adolescent girls who were in the early stage of adolescents than those who were in late adolescent age. This might be due to the increased growth spurt during the early adolescent stage as compared to late adolescent stage with a sudden increase of height in the early adolescents than late adolescents. Findings from Tigray, Ethiopia [4, 39, 46] and Belgaum and Karnataka, India [47, 48] have reported similar results with the present study.

Regarding stunting, the odds of stunting was around 1.96 times higher among adolescent girls who were in the early stage of adolescent period than those who were late adolescents. This finding is consistent with other studies conducted in five districts of Amhara region, Ethiopia [49], the baseline national nutrition survey [22], and rural community of Tigray, Ethiopia [39], which showed that prevalence and severity of stunting have been found to decrease with age. This might be due to the fact that inadequate nutrient intake besides increased requirement during early adolescent’s faster growth period and those early adolescents might be more affected by undernutrition than the older adolescents in the current study. However, a contradict finding that has been reported from Somalia region, Ethiopia was the present result [23].

The odds of stunting among adolescent girls who did not start menstruation early were 2.42 times more likely to be stunted as compared with adolescent girls who started menstruation late. This result is in line with the findings of studies done in Adwa, Ethiopia [4], Goba town, Ethiopia [50], and Western Kenya [31] which indicated a negative association between stunting with sexual maturity. This might be explained by the fact that starting menstruation coincides with the adolescent growth spurt. Delay in menstruation in stunted adolescents shows the opportunity for catch-up growth as stunting delay menarche [4, 39].

The odds of stunting among adolescent girls who had no home latrine were 3.26 times more likely to be stunted as compared with those adolescent girls who had had home latrine. This might be explained by the fact that those who have home latrine may have used it properly and they could not be affected by communicable diseases easily; as a result, they become healthy. Whereas those who do not have home latrine, they may defecate in the opened field and may be easily affected by communicable diseases; as a result growth will be interrupted and leads to stunting. Previous studies done in the rural community of Tigray, Ethiopia [39] and Tehuledere District, Ethiopia [51] showed that lack of home latrine was a predictor of stunting in adolescents.

The odds of stunting among adolescent girls from food-insecure households were 2.88 times more likely to be stunted than adolescent girls from food secured households. This indicate that the presence of chronic food insecurity leads to stunting because of chronic undernutrition and might be one of the important determinant of chronic nutritional insult in adolescent girls. The finding was in agreement with other studies conducted in Mini EDHS report and Tigray, Ethiopia [4, 44] and five districts of Amhara region, Ethiopia [49] where food insecurity is negatively associated with the linear growth of adolescents.

## Limitations of the study

The study involved a single cross-sectional design. Hence, causal inference might not be strong.

Recall and reporting bias might also affect for dietary diversity & food frequency questions. Therefore, further studies combined both quantitative and qualitative approach might be necessary for better understanding of undernutrition in the community.

## Conclusions

This study revealed that the overall prevalence of thinness and stunting were found to be 15.8% (95% CI 13.3–18.5%) and 26.6% (95% CI 23.5–29.9%) in the study area, respectively. This result indicated that thinness and stunting among the adolescent girls are public health problems in the study area according to the WHO, cutoff values for public health significance.

The independent predictor significantly associated with thinness was being early adolescents’ age while the independent predictors significantly associated with stunting were being early adolescents’ age, household food insecure, menstruation status, and availability of home latrine. A comprehensive strategy such as nutrition education, improving household economy through income-generating activities, personal and environmental hygiene practices are recommended. Interventions are also needed to improve the nutritional status of disadvantaged adolescent girls through providing comprehensive and routine nutritional assessment and counseling services at community, school, and health facility levels before they reach conception period to break the intergenerational cycle effect of malnutrition.

## Data Availability

The datasets used during the current study are available from the corresponding author on reasonable request.

## Abbreviations

AOR: Adjusted odds ratio
BSc: B achelor of Science
MUAC: Mid upper arm circumference
PPS: Proportion to population size
SD: Standard deviation
WHO: World Health Organization
